# A retrospective study of the outcome of cesarean section for women with severe pre-eclampsia in a third world setting

**DOI:** 10.4103/1658-354X.76480

**Published:** 2011

**Authors:** Obinna V. Ajuzieogu, Humphrey Azubuike Ezike, Adaobi Obianuju Amucheazi, Jamike Enwereji

**Affiliations:** *Department of Anaesthesia, University of Nigeria Teaching Hospital, Enugu, Nigeria*; 1 *Department of Obstetrics and Gynaecology, University of Nigeria Teaching Hospital, Enugu, Nigeria*

**Keywords:** *Cesarean section*, *developing country*, *general anesthesia*, *maternal/perinatal mortality*, *severe pre-eclampsia*, *spinal anesthesia*

## Abstract

**Objective:**

To compare the outcome of subarachnoid block (spinal anesthesia) and general anesthesia in Cesarean delivery for women with severe pre-eclampsia.

**Methods:**

A retrospective study of women with severe pre-eclampsia requiring Cesarean section from January 2005 to June 2009 was carried out. Maternal age, parity, gestational age at delivery, booking status, Apgar scores, maternal and perinatal mortality of the sub-arachnoid block group were compared with those of general anesthesia group using χ^2^, Student t-test and Fischer exact test.

**Results:**

There were no significant difference between the two groups in overall maternal mortality (5.4% vs. 11.9%, *P*=0.5) and perinatal mortality (2.7% vs. 11.9%, *P*=0.15). The general anesthesia group had significantly more birth asphyxia than the spinal group (55.9% vs. 27.0%, *P*=0.0006).

**Conclusion:**

There was no significant difference in the maternal and perinatal mortality outcome of cesarean delivery between women with severe pre-eclampsia who had regional anesthesia and those that had general anesthesia. There was significantly higher proportion of birth asphyxia in babies of women who received general anesthesia.

## INTRODUCTION

Pre-eclampsia is a major cause of maternal and perinatal mortality worldwide. While the symptoms and complications of pre-eclampsia are well known and far-reaching, the exact etiology remains unknown.[1]

Women with pre-eclampsia have an increased rate of cesarean section consequent upon the high incidence of intrauterine growth restriction, fetal distress and prematurity.[2] Cesarean section on the other hand increases the risk of cardiopulmonary morbidity associated with pre-eclampsia.[3] This is due to the altered hemodynamics in women with pre-eclampsia.[4] This risk is present with both spinal and general anesthesia.

The optimal anesthetic method for Cesarean section for women with pre-eclampsia remains unsettled. However, several studies have demonstrated the safety of sub-arachnoid block (spinal), epidural and combined sub-arachnoid block-epidural anesthesia for Cesarean section in women with pre eclampsia.[5–8] Studies comparing general anesthesia with other anesthetic methods are scarce. A literature search identified only one study which included general anesthesia in its comparative analysis.[9] This is understandable as most of these studies took place in developed countries where epidural and combined spinal-epidural anesthesia are routinely used for Cesarean section for pre-eclamptics. On the contrary, physicians in most developing countries like Nigeria are still restricted to either sub-arachnoid block or general anesthesia. This is due to the high cost and unavailability of epidural sets and scarcity of personnel with the requisite skills for epidural anesthesia.[10] This underscores the need for studies to compare the outcome of Cesarean section using sub-arachnoid block and general anesthesia as this will help physicians practicing in developing countries in decision-making.

This study compared the outcome of Cesarean section for pre-eclampsia using sub-arachnoid block and general anesthesia.

## METHOD

### Setting

This study was carried out at the University of Nigeria teaching hospital in Enugu, Nigeria. Sub-arachnoid block is usually done with 0.5% bupivacaine. For general anesthesia, rapid sequence induction with Sellick’s maneuver and a relaxant technique are used. Sodium thiopentone 4-6 mg/kg and suxamethonium 1-2 mg/kg are used for induction and endotracheal intubation. Anesthesia is maintained with pancuronium, halothane and oxygen/nitrous oxide.

### Data collection

The records of all women who had Cesarean section for severe pre-eclampsia from January 2005 to June 2009 were retrieved. Data on maternal age, parity, gestational age at delivery, booking status, Apgar scores, maternal mortality and perinatal mortality were extracted.

### Data analysis

The subjects were classified into two categories: Group A was patients that had sub-arachnoid block while group B comprised of patients that had general anesthesia.

The background characteristics and outcomes were compared between the two groups using χ^2^, Student t-test and Fischer exact test as appropriate, using SPSS version 10.0 statistical software. Differences were considered significant if *P*<0.05.

### Exclusion criteria

Women with mild pre-eclampsia, other medical disorders in pregnancy, multiple pregnancies, gestational age less than 32 weeks, cases of eclampsia and cases of failed sub-arachnoid block that were reverted to general anesthesia were excluded from the comparative analysis.

### Definitions

- Severe pre-eclampsia: Systolic blood pressure ≥ 160 mmHg and/or diastolic blood pressure ≥ 110 mmHg with ≥ 2+ of Proteinuria on dipstix urinalysis.
- Booked: women who received antenatal care at the study center.

## RESULTS

A total of 1156 Cesarean sections were done during the study period out of which 116 (10.0%) were for severe pre-eclampsia. Twenty cases were excluded based on the exclusion criteria leaving 96 cases for the comparative analysis.

### Type of anesthesia

Thirty-seven (38.5%) were sub-arachnoid block (Group A) and 59 (61.5%) were general anesthesia (Group B).

### Type of cesarean section

Twenty-six (70.3%) in group A were elective cases and 11 (29.7%) were emergencies. The corresponding figures in group B were 40 (67.8%) and 19 (32.2%), respectively. The difference was not statistically significant (*P*= 0.8).

### Indications for cesarean section

The indications for Cesarean section were severe pre-eclampsia with the following conditions: unfavourable cervix, previous Cesarean section, bad obstetric history, fetal distress, failed induction of labor and intra-uterine growth restriction. The distribution is shown in Table 1.

**Table 1:** Indications for cesarean section in 96 Nigerian women with severe pre-eclampsia

Indication	Group A N=37	Group B N=59	Total N=96
Severe pre-eclampsia with unfavourable cervix	30 (81.1)	41 (69.5)	71 (74.0)
Severe pre-eclampsia with previous C/S	4 (10.8)	7 (11.9)	11 (11.5)
Severe pre-eclampsia with bad obstetric history	1 (2.7)	4 (6.8)	5 (5.2)
Severe pre-eclampsia with prolonged infertility	1 (2.7)	3 (5.1)	4 (4.2)
Severe pre-eclampsia with fetal distress	0 (0.0)	2 (3.4)	2 (2.1)
Severe pre-eclampsia with failed induction of labour	0 (0.0)	2 (3.4)	2 (2.1)
Severe pre-eclampsia with IUGR	1 (2.7)	0 (0.0)	1 (1.0)
Total	37 (100)	59 (100)	96 (100)

Figures in parentheses are in percentage, C/S = Cesarean section, IUGR = Intrauterine growth restriction

### Booking status

Thirty-two (86.5%) in group A and 48 (81.4%) in group B were booked patients. The difference was not significant.

### Age

The mean ages for the patients were 31±1.4 (Range: 20-37) years and 29±2.1 (Range: 21-36) years for groups A and B, respectively. The difference was significant (*P*=0.0001).

### Gestational age at delivery

The mean gestational age at delivery was 36±1.6 (Range: 34-39) weeks for group A and 36±1.2 (Range: 34-40) weeks. The difference was not significant (*P*=1.0).

### Parity

Thirteen (35.1%) in group A were nulliparous. The corresponding figure in group B was 22 (37.3%). The difference was not significant (*P*=0.8).

Table 2 summarises the background characteristics of the two groups.

**Table 2:** Background demographics

Characteristics	Spinal anesthesia (N = 37)	General anesthesia (N = 59)	*P* value
Mean maternal age	31±1.4	29±2.1	0.0001
Mean gestational age at delivery	36±1.6	36±1.2	1
Booked patients	32(86.5)	48(81.4)	0.5
Nulliparity	13(35.1)	22(37.3)	0.8

Figures in parentheses are in percentage

### Maternal mortality

Two (5.4%) maternal deaths were recorded in group A and 7 (11.9%) in group B. The difference was not significant (*P*=0.5).

### Cause of maternal death

Anesthetic complications were responsible for 1 (2.7%) maternal death in group A and 5 (8.5%) in group B. The difference was not significant. The anesthetic complication in group A was severe hypotension that did not respond to resuscitatory measures while those in group B were wrong intubations (N=2), Mendelson’s syndrome (N=1), unexplained drug reactions (N=2).

One (2.7%) maternal death in group A and 2(3.4%) were caused by pulmonary edema. The difference was not significant.

### Apgar scores < 7 at 1 minute

Ten (27.0%) babies in group A had Apgar scores less than 7 at 1 minute. The corresponding figure in group B was 33 (55.9%). The difference was significant.

### Apgar scores < 7 at 5 minutes

Five (13.5%) babies in group A and 21 (35.6%) in group B had Apgar scores less than 7 at 5 minutes. The difference was significant.

### Perinatal mortality

One (2.7%) perinatal death were recorded in group A and 7 (11.9%) in group B. The difference was not significant.

Table 3 summarized the outcome of delivery in the two groups.

**Table 3:** Outcome of delivery

Outcome	Spinal anesthesia (N = 37)	General anesthesia (N = 59)	*P* value
Apgar score <7 at 1 minute	10 (27.0)	33 (55.9)	0.006
Apgar score <7 at 5 minute	5 (13.5)	21 (35.6)	0.02
Perinatal mortality	1 (2.7)	7 (11.9)	0.15
Maternal mortality	2 (5.4)	7 (11.9)	0.5
Maternal deaths from anesthetic complications	1 (2.7)	5 (8.5)	0.4

Figures in parentheses are in percentage

## DISCUSSION

Pre-eclampsia is a major cause of maternal mortality and morbidity, and fetal loss worldwide, but particularly in the third world. Anesthetists may be required to assist with pain management in labor, to provide anesthesia for Cesarean section and to assist in the Intensive Care Management of life-threatening complications which may arise from this condition.

Severe pre-eclampsia is defined as any one of the following occurring after the 20^th^ week of pregnancy: (i) severe hypertension (systolic blood pressure > 160 mmHg or diastolic blood pressure > 110 mmHg); (ii) proteinuria > 5 g per 24 h; (iii) oliguria < 400 ml per 24 h; (iv) cerebral irritability or visual disturbances; (v) epigastric or right upper quadrant pain (liver capsule distension); or (vi) pulmonary edema.[10]

There was no significant difference in the background maternal characteristics of the patients studied except for the mean maternal age. The exclusion criteria helped to eliminate confounding factors in the comparative analysis. The incidence of Cesarean section for pre-eclampsia (10%) was in keeping with the worldwide incidence of pre-eclampsia.[11]

The proportion of patients that received sub-arachnoid block showed a remarkable improvement to 38.5% in this study from 7.8 % reported by researchers from the same practice environment during the period 1998-2002.[12] This dramatic increase in the use of spinal anesthesia resulted from the documented safety of sub-arachnoid block for pre-eclamptics undergoing Cesarean section. The absence of studies from this environment demonstrating advantage of sub-arachnoid block over general anesthesia for severe pre-eclamptics may be a contributory factor to the preponderance of general anesthesia noted in this study.

The proportion of maternal deaths from anesthetic complications was not significantly different between both groups. An earlier study from a developed country setting compared general anesthesia, epidural anesthesia and combined sub-arachnoid block-epidural anesthesia for women with severe pre-eclampsia and found no difference in the outcomes.[9] Significantly more babies with Apgar scores less than 7 at 1 and 5 minutes were recorded in the general anesthesia group than in the sub-arachnoid block group. However, the perinatal mortality was not significantly different between both groups.

## CONCLUSION

The findings in this study support the previous studies which showed no significant difference in the maternal and perinatal mortality outcome of Cesarean delivery between women with severe pre-eclampsia who had regional and those that had general anesthesia. However, there was significantly higher proportion of birth asphyxia in women who received general anesthesia.
